# The Proteomics-Based Stratification of Obese Subjects Allows for a Second Selective Level Beyond Gender Classification

**DOI:** 10.3390/ijms27114678

**Published:** 2026-05-22

**Authors:** Raffaello Viganò, Jonica Campolo, Francesca Brambilla, Dario Di Silvestre, Ettore Corradi, Marina Parolini, Cinzia Dellanoce, Patrizia Tarlarini, Paolo Iadarola, Francesco Scaglione, Pierluigi Mauri

**Affiliations:** 1 Institute for Biomedical Technologies, National Research Council (ITB-CNR), Via Fratelli Cervi 93, 20054 Segrate, Italy; raffaello.vigano@itb.cnr.it (R.V.); francesca.brambilla@itb.cnr.it (F.B.); dario.disilvestre@itb.cnr.it (D.D.S.); 2 CNR Institute of Clinical Physiology (IFC-CNR), ASST GOM Niguarda, Piazza dell’Ospedale Maggiore 3, 20162 Milan, Italy; jonica.campolo@cnr.it (J.C.); marina.parolini@cnr.it (M.P.); cinziacarla.dellanoce@cnr.it (C.D.); francesco.scaglione@unimi.it (F.S.); 3 Clinical Nutritional Unit, ASST GOM Niguarda, Piazza dell’Ospedale Maggiore 3, 20162 Milan, Italy; ettore.corradi@ospedaleniguarda.it (E.C.); patrizia.tarlarini@ospedaleniguarda.it (P.T.); 4 Department of Biology and Biotecnologies “Lazzaro Spallanzani”, University of Pavia, Via Adolfo Ferrata 9, 27100 Pavia, Italy; paolo.iadarola@unipv.it; 5 Department of Chemical-Clinical and Microbiological Analyses, ASST GOM Niguarda, Piazza dell’Ospedale Maggiore 3, 20162 Milan, Italy; 6 Institute of Endotypes in Oncology Metabolism and Immunology “G. Salvatore” (IEOMI-CNR), 80131 Naples, Italy

**Keywords:** obesity, proteomics, LC-MS/MS, systems biology, molecular profiles

## Abstract

Obesity is a major global health challenge characterized by chronic low-grade inflammation, oxidative stress, and an increased risk of cardiometabolic disorders. Although sex-related differences in inflammatory and redox biomarkers have been reported in obese populations, the molecular mechanisms underlying this heterogeneity remain incompletely understood. In this study, we applied a proteomics-based approach to investigate urinary extracellular vesicles from 45 obese individuals (BMI 30–40 kg/m^2^; age 50–70 years) in order to identify molecular signatures associated with metabolic dysregulation. Shotgun proteomics analysis performed by nanoLC–MS/MS enabled the identification of 3822 proteins. Hierarchical clustering of proteomic profiles revealed two distinct molecular groups, predominantly enriched in males (Group I) and females (Group II). Label-free quantitative analysis identified 466 differentially abundant proteins between the two clusters. Functional enrichment analysis highlighted pathways associated with immune response, metabolic regulation, and redox homeostasis, including glycolysis/gluconeogenesis, lysosome activity, leukocyte transendothelial migration, and glutathione, cysteine and methionine metabolism. Notably, proteins related to ferroptosis were enriched, suggesting the involvement of iron-dependent oxidative cell death mechanisms in the metabolic imbalance observed in a subset of subjects. Furthermore, the non-enzymatic glycosylation of urinary proteins was significantly higher in Group I compared with Group II (*p* = 0.0002), indicating increased formation of advanced glycation products in individuals with a more pronounced pro-oxidant state. Preliminary follow-up data suggested a higher incidence of pathological events, including cardiovascular complications, among individuals belonging to Group I. Overall, these findings demonstrate that urinary proteomic profiling can identify distinct molecular phenotypes among obese individuals and highlight oxidative stress, ferroptosis, and protein glycation as potential determinants of metabolic vulnerability, supporting the use of non-invasive proteomic approaches for improved risk stratification in obesity.

## 1. Introduction

Over the past four decades, obesity has escalated to pandemic levels worldwide, presenting a major public health challenge [1].

This rise is largely driven by behavioral and environmental factors, including sedentary lifestyles and the widespread availability of energy-dense, nutrient-poor foods.

Physiologically, chronic energy intake exceeding expenditure leads to the storage of triglycerides in adipose tissue, which expands through adipocyte hypertrophy, contributing to progressive increases in body weight and metabolic dysfunction.

Weight gain, particularly visceral fat accumulation and reduced physical activity, impair the function of adipose and muscle tissues, promoting insulin resistance and systemic inflammation [2]. This inflammatory state plays a central role in the pathogenesis of numerous obesity-related comorbidities, such as type 2 diabetes, cardiovascular disease, non-alcoholic fatty liver disease, and certain cancers [3].

In obesity, the overproduction of oxidative stress and inflammatory biomarkers occurs in parallel with a reduction in antioxidant and anti-inflammatory mediators. These imbalances are part of the cellular mechanisms that disrupt metabolic homeostasis. In a previous work, Campolo J. et al. found an increment of the total and oxidized aminothiol forms, which are prooxidant mediators able to produce free radicals, and the depletion of reduced GSH, the most important antioxidant present in our organism, in men compared to women despite comparable levels of visceral adiposity (VAI) and insulin resistance (HOMA). Furthermore, gender-specific alterations in vitamin D concentrations and adipomyokines profile were also observed [4].

Despite well-documented evidence of oxidative imbalance in obesity, the biological pathways underlying gender-related differences in redox balance and inflammation—and their links to cardiometabolic risk—remain; however, they are incompletely understood and warrant further investigation. A more in-depth knowledge from a holistic perspective on changes induced by obesity on metabolic and functional pathways could open new outlooks in treatment of this condition, especially in a prognostic way. In particular, proteomics could play a pivotal role in unveiling altered proteins and pathways that underline systemic pro-inflammatory and pro-oxidant status. In the last years, a few studies have highlighted how the proteome is consistently altered in several tissues/biofluid in obese subjects from a systemic point of view [5,6,7], and in addition, experimental specimens with minimal invasive sampling like urines were applied in proteomics to describe and characterize not only renal or urinary system pathologies, but also systemic diseases like coronary artery disease, hypertension, and respiratory diseases [8,9,10]. To the best of our knowledge, only few previous studies have tried to describe the molecular differences among obese patients using a proteomic approach [11], and none of them employed urine samples to characterize and stratify potential underlying mechanisms. In this context, our study aimed to investigate molecular variations in individuals predisposed to metabolic disorders, already known to exhibit sex-specific differences in inflammatory markers [4], to better define the molecular changes associated with this distinct phenotype.

## 2. Results

### 2.1. Enrolled Population

We enrolled 45 patients with a mean age of 60 ± 5 years, consisting of 25 men and 20 post-menopausal women. The clinical and anthropometrical characteristics, and the routine biochemical profile are depicted in Table 1.

### 2.2. LC-MS/MS Analysis

#### 2.2.1. Proteome Profiling

Proteomics analysis by means of nano-LC-MS/MS-based shotgun bottom–up was performed on the urine’s samples of 45 different subjects (21 males and 24 females), producing two technical replicates per each subject, except for 2 subjects in which only one replicate was obtained. This approach led to the identification of 3822 distinct proteins from a total of 88 protein lists (including biological and technical replicates), merged to obtain a single master protein matrix. The complete list of identified proteins is reported in Table S1 of the Supplementary Material. These results confirm the effectiveness of proteomics approaches in covering the urine proteome widely, through repeated analyses of biological and technical replicates of homogeneously stratified subjects.

#### 2.2.2. Hierarchical Clustering

**Figure 1:**
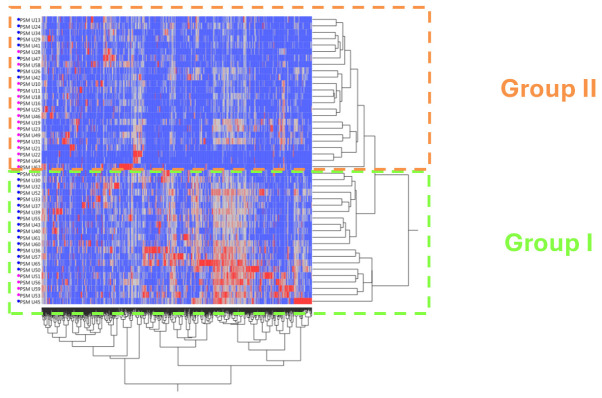
Hierarchical clustering (HC) analysis of the 45 subjects (blue circle: male; pink circle: female), obtained with a relative abundance level of the 3822 identified proteins. For each protein, the relative abundance in each subject value is represented by a color from blue to red, according to increasing abundances. Main groups identified on the base of HC are depicted in light green (Group I) and orange (Group II), while subjects’ sex is represented by a blue circle (males, 21 subjects) and a pink circle (females, 24 subjects) next to the subject identifier.

The obtained urine protein profiles (3822 proteins) were used to evaluate the potential subgrouping of subjects through Hierarchical Clustering (HC). Specifically, technical replicates normalized protein lists from the same subjects were mediated to obtain a single protein list for each of the 45 analyzed subjects, and Peptide Spectral Matches (PSMs) attributed to each protein were used to perform a cluster analysis by means of JMP software (version 15.2). The heatmap resulting from HC showed stratification in two main groups: one predominantly grouped male subjects (Group I, 17 out of 21 group subjects, corresponding to the 81% of subjects, and representing the 68% of overall male subjects), and the other evidenced a female majority, Group II (16 out of 24 group subjects, corresponding to the 67% and representing the 80% of overall female subjects) (Figure 1).

#### 2.2.3. Differentially Abundant Proteins (DAPs)

The extraction of the heatmap-related descriptors (*p*-value < 0.05) and label-free quantification analysis by means of MaProMa platform evidenced 466 proteins classified as differentially abundant (DAPs) that are listed in Table S2 of the Supplementary Material. Relative abundances of these proteins were calculated by means of DAve (Differential Average Index) and DCI (Differential Confidence Index) indexes, obtained as previously described [12] and filtered at |0.2| and |5|, respectively. Relative abundances of identified DAPs were employed to perform a principal component analysis (Figure S1) that confirmed separation in two clusters, thus enforcing and underlining the robustness of the HC obtained with the total protein profiles.

#### 2.2.4. Pathway Enrichment

**Figure 2:**
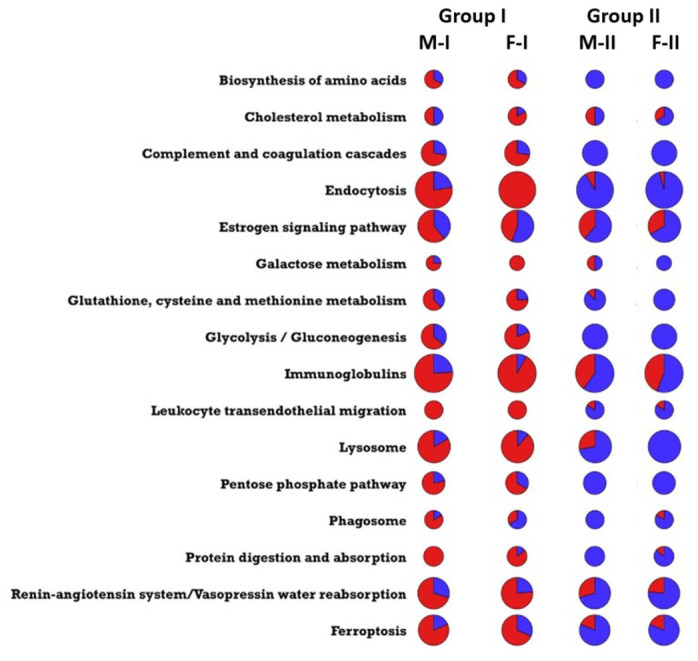
Protein expression per functional pathway in M-I, F-I, M-II and F-II. Each bubble size is proportional to the number of proteins in a specific pathway (KEGG); the blue area is proportional to the number of low abundant proteins, while the red area is proportional to highly abundant proteins.

The 466 DAPs extracted from hierarchical clustering were submitted to STRING database to evaluate possible functional association networks among the considered proteins. Considering the submitted list, from STRING database the enriched functional pathways based on KEGG database annotation were extracted and, after manual revision, 15 different functional pathways resulted enriched in the descriptors protein list. The KEGG functional terms that resulted enriched after this analysis were as follows: “Immunoglobulins”, “Endocytosis”, “Lysosome”, “Complement and coagulation cascades”, “Leukocyte transendothelial migration”, “Phagosome”, “Renin-angiotensin system and vasopressin water reabsorption”, “Glutathione, cysteine and methionine metabolism”, “Estrogen signaling pathway”, “Glycolisis/gluconeogenesis”, “Pentose phosphate pathway”, “protein digestion and absorption”, “Biosynthesis of amino acids”, “Cholesterol metabolism” and “Galactose metabolism”. To better understand the relative behavior of each pathway between the two groups identified through Linear Discriminant Analysis (LDA), for each male and female sub-population a Protein-Protein Interaction (PPI) network was constructed by means of Cytoscape software (version 3.8) (Figures S2–S5); the network is composed by DAPs, representing the nodes of the net, connected among each other by edges that indicate either physical or functional interaction and grouped by functional pathway. A color graduation scale, from blue to red, is attributed to each node according to the relative abundance of a specific protein in sub-populations: the highest value of Peptide Spectral Matches (PSMs) associated with the same protein in M-Gr. I, M-Gr. II, F-Gr. I and F-Gr. II are set to 100 (red node), while the other PSMs values are expressed as a relative percentage and, consequently, represented as the corresponding colors. To resume the PPI networks, a series of pie charts named “Bubble chart” was elaborated (Figure 2). Each pathway is represented as a circle for each sub-population, where the diameter is proportional to the total number of proteins belonging to each pathway. The blue area of the bubble is proportional to decreased abundance (or low abundant) proteins while the red area to increased abundance (or highly abundant) proteins within each sub-population. To classify a protein as up represented or down represented, the PSMs values in each group were considered: the highest value among them was set as 100, and the others were expressed as relative values. To consider a protein as increased in abundance, the relative PSMs value in a specific sub-population had to be equal or higher than the 50% of the reference value; otherwise, the protein was classified as decreased in its abundance.

As shown in Figure 2, the expression of proteins belonging to the enriched KEGG pathways seems to be independent from the subjects’ gender, being strongly related to the proteomic-identified groups instead. Group I shows a general up-representation of considered protein species if compared to Group II. Of note, pathways related to both cellular and systemic redox balance, like “Ferroptosis” or “Glutathione, Cysteine and Methionine Metabolism”, resulted as up represented in Group I after our analysis. The same trend is shown by “Phagosome”, and a proteostasis-related pathway like “Lysosome”. These results taken together suggest that in Group I subjects the processes related to inflammation, such as glutathione, cysteine and methionine metabolism and ferroptosis, and subsequent redox stress are strongly triggered in comparison to Group II; these findings seem to be a reflex of an inflammation-prone state of Group I subjects, which could request an intense activity of antioxidant molecules like GSH and cysteine and an effective scavenging of ROS by several different systems.

Furthermore, remarkable differences can be observed between Group I and II also in cell metabolism-related annotations. Pathways like “Glycolysis/Gluconeogenesis”, “Biosynthesis of Aminoacids” and “Galactose Metabolism” seem to be strongly represented in Group I.

This behavior could be a symptom of a more dysregulated metabolism in Group I subjects, with a more pronounced shift towards glycolysis, that was already reported for patients with obesity in other samples by proteomics approaches.

#### 2.2.5. FerrDB Enrichment Analysis

**Figure 3:**
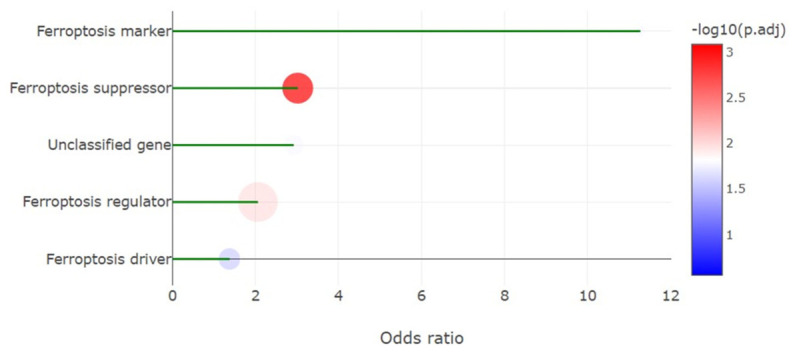
Results of FerrDB enrichment. Among the 466 identified DAPs, 16 were annotated as “Ferroptosis Driver”, 39 as “Ferroptosis Suppressor” and 6 as “Ferroptosis Marker”. The length of the green bar for each enriched term represents the magnitude of the odds ratio enrichment, while the circle at the end represents, with its color, the −log10 (p.adj), according to the scale depicted in the figure.

Interestingly, the STRING and KEGG enrichment analysis described above resulted in sixteen proteins annotated for the functional pathway “Ferroptosis” (Figures S2–S5). In terms of number of proteins attributed to a specific pathway, ferroptosis was the sixth most represented, after “Immunoglobulins”, “Endocytosis”, “Lysosome”, “Estrogen Signaling Pathway” and “Renin-angiotensin system/Vasopressin water reabsorption”. Given the emerging role that ferroptosis gained in the last years, especially in obesity [13,14], and the interplay that ferroptosis has with oxidative stress [15], our attention was captured to ask whether other proteins among the differentially abundant we identified could belong to this pathway. Therefore, the 466 identified DAPs were used also to perform an enrichment analysis on FerrDB v2 [16], and evaluate if other proteins among the differentially abundant ones could be related to ferroptosis. Interestingly, among the 466 proteins considered, two annotations were enriched: “Ferroptosis Driver” (16 proteins, adj. *p*-value = 0.002) “Ferroptosis Suppressor” (39 proteins annotated, *adj. p*-value = 0.0008) and “Ferroptosis Marker”, with six proteins annotated (*p*-value < 0.018). These results are shown in Figure 3. The List of proteins identified as “Ferroptosis Driver”, “Ferroptosis Suppressor” and “Ferroptosis Marker” is available in Table S4, in the Supplementary Material.

These findings corroborate the results obtained from the systems biology analysis and underscore the involvement of ferroptosis in the pathophysiology of dysmetabolism among obese patients. Ferroptosis is a programmed cell death mechanism alternative to apoptosis and related to ROS production, that can promote this mechanism through peroxidation of lipids. In this context, patients of Group I, that show a greater expression of proteins related to ferroptosis, may be under a pro-ferroptotic condition if compared to Group II subjects. Thus, this condition could require an increased activity of antioxidant molecules, like GSH and cysteine, coupled with other systems of scavenging of oxidative stress related to ferroptosis, like the GSH/GPX4 axis. Of course, since these results come from a bioinformatic prediction, they represent an in silico predictive evaluation that should be confirmed by further and more specific validation.

#### 2.2.6. Non-Enzymatic Glycosylation Search

Apart from protein identification, a search for non-enzymatic glycosylation derived peptides was performed. This analysis allowed for the identification of 3208 different peptides modified on R residues, associated to 2092 different proteins, and 2403 different peptides modified on K, attributed to 2403 different proteins. The glycosilation adducts involving arginine that were considered were as follows: Nε-[5-(2,3,4-Trihydroxybutyl)-5-hydro-4imidazolon-2-yl]ornithine (3-DG-H1), Tetrahydropyrimidine (THP), Imidazolone B (IB), Argpyrimidine (ArgP), Nε-(5-Hydro-5-methyl-4-imidazolon-2-yl)ornithine (MG-H1) and Nε-(5-Hydro-4-imidazolon-2-yl)ornithine (G-H1); the adducts involving lysine considered were: Fructosyl-lysine (FL), Fructosyl-lysine-H_2_O (FL-1H_2_O), Fructosyl-lysine-2H_2_O (FL-2H_2_O), Nε-Carboxyethyl-lysine (CEL), Nε-Carboxymethyl-lysine (CML) and Pyrraline (Pyr); eventually, it was considered also an adduct involving lysine or arginine, namely 1-Alkyl-2-formyl-3,4-glycosyl-pyrrole (AFGP).

After identifying two different main groups of subjects resulting from the HC, some clinical, physiological and biochemical parameters measured at the enrolment or measured with proteomic analysis were considered to evaluate if they could fit with the proteomic clustering, and thus correlate a molecular profiling obtained with nLC-MS/MS with phenotypical parameters, commonly evaluated in clinical routine.

**Figure 4:**
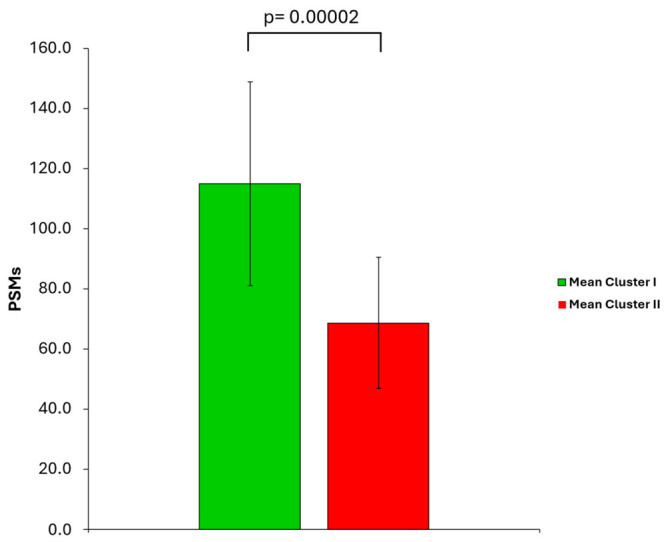
Non-enzymatic glycosylation levels in proteomics Group I (green) and II (red). The lower non-enzymatic glycation level in Group II is statistically significant (*p* = 0.00002).

**Figure 5:**
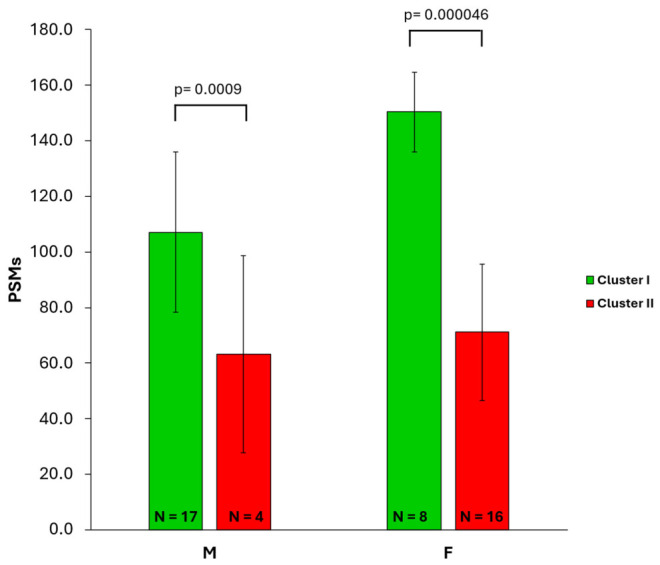
Non-enzymatic glycosylation levels in male and female sub-populations of Group I (green) and II (red). The lower non-enzymatic glycosylation level in Group II is maintained both for males (M) (*p* = 0.0009) and females (F) (*p* = 0.000046).

Firstly, it was examined if the glycosylation levels may differ between the two groups considered. Interestingly, the group I showed higher average levels of glycosylation on K and R residues if compared to group II (*p* (*t*-test) = 0.000002) (Figure 4). Of note, the statistical significance was maintained comparing male and female sub-populations in the two groups: males Group I (M-I) vs. males group II (M-II) (*p* = 0.0009) and females Group I (F-I) vs. females Group II (F-II) (*p* = 0.000046) (Figure 5).

#### 2.2.7. Comparison of Clinical and Biochemical Parameters Between Proteomics Groups

Clinical and biochemical characteristics were compared between proteomic groups in all patients. The results are reported in Table S3. In Group I we found a prevalence of male gender, while in Group II we observed significantly higher fat body mass and VAI. It is known that lean and fat body mass are related to gender, and our results are consistent with literature.

The differences observed between Groups I and II suggest that sex—predominantly male in Group I and female in Group II—may play a crucial role in shaping the key metabolic characteristics and behaviors of this population, as previously reported by Campolo et al., 2021 [4].

#### 2.2.8. Follow up of the Subjects

After 6.0 ± 0.3 years of the enrolment for this study, subjects included in this cohort were contacted to undergo a follow-up survey. Considering the 45 subjects whose urines were analyzed with nano LC-MS/MS, 13 subjects responded to the survey: 5 belong to proteomic Group I and 8 belong to Group II. Of these 13 subjects, only 4 did not report a pathology event that emerged after the enrolment; notably, three of them belong to Group II. This fact means that 4/5 (80%) Group I subjects that responded to the survey presented a pathology after the enrolment, while for Group II 5/8 (62.5%) subjects reported a pathology after enrolment. Notably, considering the serious cardiovascular events (i.e., myocardial infarction, coronary disease, etc.), 2 subjects reported an event; these two subjects belong to the proteomic Group I, representing the 40% of total responders to the survey for this group. Interestingly, none of these events were reported by Group II subjects. Although these results are very partial and limited by the low number of survey responders and thus represent only an exploratory observation that should not be over-interpreted, this trend suggests that proteomic Group I could be more prone to developing some severe disease state, with a higher incidence of cardiovascular pathologies due to the cohort characteristics.

## 3. Discussion

The present study concerns the proteomics-based investigation of urine samples from 45 obese subjects, selected according to specific enrollment criteria; specifically, this approach enabled unbiased stratification into two groups (Group I and Group II). Notably, these groups resulted in good agreement with previous findings obtained from the same cohort [4], where the authors stratified subjects by gender a priori; in fact, our Groups I and II are populated by males and females, respectively. The two clusters differed in several clinical parameters, including lean body mass, fat body mass, and visceral adiposity index, as for male gender, that resulted as prevalent in Group I, while the female gender was enriched in Group II (Table S3). However, our unbiased stratification underlined the presence of four females in the male-based group (I), and eight male subjects in the female-based group (II). These observations indicate that, although sex distribution differed significantly between groups gender alone does not fully account for the molecular stratification.

The protein–protein interaction network of differentially abundant proteins (DAPs) enabled the identification of functional modules altered between Group I and Group II. Specifically, pathways such as glycolysis, lysosomal function, leukocyte transendothelial migration endocytosis, ferroptosis and the renin–angiotensin system were overexpressed in Group I relative to Group II. Furthermore, proteomics-based evidence demonstrates that male and female subpopulations within a given proteomic cluster display minimal intra-group differences, while substantial differences are observed when comparing individuals of the same sex across the two proteomic groups.

Upregulation of glycolysis has been associated with M1 macrophage polarization in obesity and with a consequent pro-inflammatory phenotype [17,18]. Johnson et al. further suggested that increased glycolytic flux and mitochondrial disruption may be driven by high fructose intake typical of Western diets [19]. These insights support the notion that glycolytic activity may serve as a “sensor” of inflammation-prone states, even within a demographically homogeneous cohort. Moreover, lysosomal dysfunction has been linked to several obesity-related conditions, including cardiovascular and cerebrovascular diseases [20,21], as well as to M1 macrophage polarization under diabetic conditions, reflecting enhanced inflammatory burden [22]. In this context, generalized upregulation of molecules promoting leukocyte migration may signal a chronic low-grade inflammatory status, potentially more pronounced in the male-predominant Group I. This speculation is consistent with Weinstock et al., who emphasized the role of various leukocyte types—such as macrophages, monocytes, dendritic cells, and neutrophils—in sustaining inflammatory processes within white adipose tissue [23]. Additionally, mitochondrial impairment in obesity can exacerbate oxidative stress, promoting chemokine production that recruits M1 macrophages and perpetuates a pro-inflammatory cycle [24]. Our data agree with this model, revealing concomitant upregulation of leukocyte migration pathways and those involved in redox homeostasis and ROS scavenging, including glutathione, cysteine and methionine metabolism, and ferroptosis.

Among the pathways differentially expressed between the two groups, ferroptosis warrants particular attention. Originally, Dixon et al. characterized ferroptosis as an iron-dependent, non-apoptotic form of cell death driven by lipid reactive oxygen species [15]. Ferroptosis is regulated by multiple mechanisms, including inhibition of the Xc^−^ amino acid antiporter, suppression of GPX4, modulation by specific acetylation-deficient p53 mutants, and regulation through mitochondrial VDAC transporters [15]. Recent studies have highlighted the relevance of ferroptosis in obesity, suggesting that dysregulated iron metabolism may trigger this pathway [25,26], underlining the central role of ROS in obesity-associated inflammation and redox imbalance. We acknowledge that in our work the link between ferroptosis and an obesity state is given only by indirect observations, but it is important to note that ferroptosis has accordingly been proposed as a potential therapeutic target for obesity-related metabolic disorders [27,28], although its direct consequences in obese subjects remain incompletely understood and warrant further investigation. Within this framework, our study provides insight into the interplay between ferroptosis and other pro-inflammatory functional annotations, revealing molecular distinctions within a demographically homogeneous cohort. Campolo et al. previously confirmed, in their broader cohort that included the subjects assessed here, a gender-driven stratification characterized by differences in blood and plasma redox markers, with males appearing more prone to pro-inflammatory and pro-oxidant states [4].

Another noteworthy observation concerns the level of non-enzymatic glycosylation of urinary proteins. Glycosylation was significantly higher in the male-predominant Group I, and this trend persisted when comparing male Group I vs. male Group II and female Group I vs. female Group II. This result is particularly interesting since the glycosylation of proteins has already been linked to reactive oxygen species (ROS) in the context of inflammation [29]. A vicious cycle appears to exist in the onset of type-2 diabetes, a disease that is due to its association with a high fat diet characterized by hyperglycemia and insulin resistance that led to oxidative stress and inflammation. Chronic elevation of free fatty acids and increased production of ROS in mitochondria also attenuate insulin secretion in pancreatic β-cells and thereby trigger the onset of type-2 diabetes [30]. In addition, the production of advanced glycation end products (AGEs), the final products of the non-enzymatic glycosylation process, arise through the Maillard reaction, both in foods and via endogenous metabolic processes [31], is enhanced under conditions such as hyperglycemia, oxidative stress, and transition metal-mediated free radical formation [32]. In obesity, elevated AGE levels can promote ROS production and upregulate pro-inflammatory cytokines such as IL-1, IL-6, and TNF-α via NF-κB activation, thereby contributing to metabolic syndrome, diabetes, and cardiovascular disease development [33]. These findings highlight the need for deeper investigation into the interrelationships between nEGs, circulating AGEs, and tissue AGE accumulation, an area that remains insufficiently understood.

Overall, our data indicates that subjects in proteomics-based Group I exhibit a more pronounced systemic pro-inflammatory and pro-oxidant state compared with Group II. Thus, our study evidenced that classification of obese individuals may be improved by incorporating oxidative status alongside gender. This interpretation is supported by preliminary follow-up questionnaire results obtained in a small subgroup, which revealed that 80% of Group I developed a pathology during a mean of 6 years follow-up, compared with 62.5% of Group II. Notably, 40% of Group I experienced severe adverse cardiovascular events, while none were observed in Group II.

Although the current results are preliminary and exploratory, they represent, to our knowledge, the first attempt to stratify a homogeneous cohort of obese individuals based on their molecular profiles and correlate them with their biochemical characteristics. Future studies aimed at addressing the limitations of this study, including validation of the results using absolute quantitative methodologies, targeting specific protein candidates and dysregulated metabolic pathways, preferably in an independent validation cohort, need to be performed to shed light on the results hereby presented. In addition, concerning the follow-up, although the limited response rate (13 of 45 subjects) precludes definitive conclusions, the observed trends support the hypothesis that increased oxidative stress in Group I may contribute to a higher risk of adverse health outcomes.

Taken together, these findings provide proof of concept for the value of proteomic analyses in the study of obese individuals using non-invasive samples, such as urines, and give a first picture of the complex molecular state of obesity that should be addressed and investigated in more detail.

## 4. Materials and Methods

### 4.1. Study Population

Subjects aged between 50–70 years, with BMI within 30–40 kg/m^2^ were enrolled in the study. The study population enrolled for proteomic analysis is a subset of participants from the Amanda Project (Regione Lombardia-CNR 2016–2018 Framework Agreement, grant 19364/RCC). This cohort consisted of healthy obese individuals with different degrees of insulin resistance and no comorbidities. Such a condition is more prevalent in middle-aged and younger elderly individuals [34]. Patients with kidney impairment (creatinine > 2.5 mg/dL), liver dysfunction (AST-ALT levels exceedingly twice the upper normal range) or active cancer were instead excluded. All participants provided written informed consent, and the study protocol was approved by the Local Ethical Committee in accordance with the principles of the Helsinki Declaration.

Participants followed a weight maintenance program that included lifestyle guidance and a diet based on the principles of the modern Mediterranean diet pyramid, promoting variety in food choices, seasonal ingredients, and the use of spices for flavor.

### 4.2. Anthropometric Measurements

Anthropometrical data (height and weight) were measured and collected for each patient and BMI value was calculated as follows: body weight (kg)/height (meters) squared. Waist circumference was measured in centimeters by taking two measurements, with the final value recorded as the average of the two.

### 4.3. Body Composition Assessment

Fat mass and lean mass were measured using air displacement plethysmography (BOD POD^®^, Cosmed, Rome, Italy), which calculates body volume by detecting pressure changes in a closed chamber. Two measurements were taken: (1) the volume of the empty chamber, and (2) the volume of the chamber with the participant inside. The participant’s volume was then determined by subtraction. Based on the participant’s body density, the relative proportions of body fat and lean mass were calculated using the Siri equation: % fat mass = [495/Density] − 450, and % lean mass = 100 − % fat mass.

### 4.4. Biological Sample Processing and Storage

Eligible subjects attended our Institute in the fasting state to perform peripheral blood collection and donate urine samples.

Blood in EDTA was centrifugated at 4000 rpm, 4 °C for 10 min in order to obtain plasma aliquots while blood collected in serum separator tubes, was kept at room temperature for 30 min to allow sample coagulation before centrifugation at 4000 rpm for 15 min.

However, urine samples were processed to obtain exosome vesicles (EVs) using specific centrifugation protocols. A first centrifugation at 16,500× *g*, for 20 min at 4 °C, to eliminate cells debris, was followed by two cycles of ultracentrifugation at 125,000× *g* for 90 min at 4 °C. The obtained pellets were frozen at −20 °C and stored until further processing for proteomic analysis.

### 4.5. Biochemical Assessment

Fasting glucose and insulin, total/HDL/LDL cholesterol, triglycerides, AST, ALT and GGT were measured by standard laboratory procedures. HOMA index, was calculated as follows: (fasting glucose mg/dL × fasting insulin microU/mL)/405.

### 4.6. Adiposity Indices

The Fatty Liver Index (FLI) is an algorithm based on BMI, waist circumference, triglycerides and gamma-glutamyl transferase and it is one of the best-validated markers for liver steatosis [35].

The Visceral Adiposity Index (VAI) is an empirical-mathematical model, gender-specific, based on simple anthropometric (BMI and waist circumference) and functional parameters (triglycerides and HDL cholesterol) indicative of fat distribution and function [36].

### 4.7. Metabolic Syndrome

The metabolic profile was assessed using a multicomponent cardiometabolic risk score, which included the following parameters: waist circumference ≥ 88 cm in women and ≥100 cm in men, systolic/diastolic blood pressure ≥ 130/85 mmHg, HDL cholesterol < 50 mg/dL in women and <40 mg/dL in men, triglycerides ≥ 150 mg/dL, and fasting plasma glucose ≥ 100 mg/dL. According to the NCEP ATP III criteria [37], subjects were classified as having metabolic syndrome if at least three of these five parameters were present.

### 4.8. Sample Preparation for LC-MS/MS

The isolated EVs from urine samples were resuspended in 0.1 M ammonium bicarbonate (Sigma-Aldrich Inc., St. Louis, MO, USA) pH 7.8, to reach an equal volume for all analyzed samples. Rapigest surfactant (Waters, Milford, MA, USA) prepared at 2% concentration in NH_4_HCO_3_ 0.1 M was added to samples, to reach a final concentration of 0.2%. The samples were then incubated for 20 min at T (°C) = 100 °C and concentrated in the Speed Vac concentrator (ThermoFisher Scientific, Waltham, MA, USA) until reaching a V = 50 µL. Protein concentration was assessed with SPN Protein Assay Kit (GBiosciences, St. Louis, MO, USA), following the manufacturer’s instructions. After quantification, 50 µg of proteins per sample were digested with trypsin (sequencing grade) (Pierce, Rockford, IL, USA), with an enxyme:substrate ratio equal to 1:50; samples were digested overnight at T (°C) = 37 °C, 400 rpm. The next day, a second digestion step with trypsin was performed for 4 h, T (°C) = 37 °C, 400 rpm. After digestion, samples were acidified with trifluoroacetic acid (Panreac Quimica, Barcelona, Spain) to reach a final pH = 2. Samples were incubated for 45 min., T (°C) = 37 °C, 400 rpm and were centrifuged at 13,000 rpm for 10 min. Peptides obtained after tryptic digestion were purified with PepClean C18 Columns (Pierce, Rockford, IL, USA) following manufacturer’s instructions. Finally, purified peptides were lyophilized and resuspended in H_2_O + 0.1% formic acid and stored at +4 °C until analyses.

### 4.9. LC-MS/MS Analysis

Each sample was analyzed in two technical replicates, injecting 0.8 µg per each run, on a LC-MS/MS platform composed as follows: Eksigent nanoLC-Ultra 2D System (Eksigent, Dublin, CA, USA) for nano liquid chromatography, coupled with a LTQ Orbitrap XL^TM^ (Thermo Fisher Scientific, San Jose, CA, USA) for MS/MS analyses. In particular, peptides were firstly trapped with a trap column (Zorbax 300 SB-C18, 0.3 i.d. × 5 mm, 5 µm, 300 Å; Agilent Technologies, Santa Clara, CA, USA) with a gradient running for 10 min. in isocratic mode at 3 µL/min, and then separated with a C18 separating column (Biobasic-C18, 0.18 i.d. × 100 mm, 5 µm, 300 Å, Thermo Fisher Scientific, San Jose, CA, USA), running the following 130 min. eluent gradient at 300 nL/min: (A) 0.1% formic acid in water; (B) 0.1% formic acid in acetonitrile; the gradient profile was 5–10% B in 3 min., 10–50% B in 94 min., 50–95% B in 13 min., 95% B for 7 min. followed by 10 min. at 5% B for column equilibration. Peptides were analyzed by tandem MS where mass spectra were recorded in positive ion mode over a 400–1600 *m*/*z* range at a 30,000 FWHM (full width at half maximum) resolution acquired with Orbitrap analyzer, followed by 5 MS/MS spectra acquired with Ion Trap analyzer, at a resolution of 5000 FWHM, generated in a data-dependent manner on the most intense ions in MS1. Activation Type was set as CID, while Normalized Collision Energy was set at 35%, and Activation Q equal to 0.250. Maximum injection time was set to 30 ms for both MS1 and MS/MS, while isolation window was set to 2 *m*/*z*. Dynamic exclusion was set equal to 30 s, and charge state for peptides to be fragmented was set from +2 to +4. AGC target was set to 5 × 10^5^ for MS1 and to 1 × 10^4^ for MS/MS. The spray capillary voltage was set at 1.6 kV, and the ion transfer capillary temperature was maintained at 220 °C.

### 4.10. Protein Identification

All data generated were searched using Proteome Discoverer 2.1 platform (ThermoFisher Scientific, Waltham, MA, USA) based on SEQUEST search engine and human protein database (70,726 entries, downloaded on March 2024 from UNIPROT website, (https://www.uniprot.org/). Trypsin was set as digestion enzyme; three missed cleavages were set at maximum; mass tolerances of 10 ppm for precursor ions and 0.05 Da for fragment ions were applied. A percolator node was used with target-decoy strategy to give final false discovery rates (FDR) at a Peptide Spectrum Matches (PSMs) level of 0.01 (strict) based on q-values, considering a maximum deltaCN of 0.05; a minimum peptide length of six amino acids and rank 1 were considered, and protein grouping and strict parsimony principle were applied. The identified protein list with PSMs values was exported in .xlsx format for further evaluation of results. PSMs values were imputed with zero in case of missing values and normalized following a global signal strategy-based normalization prior to further evaluation.

### 4.11. Non-Enzymatic Glycosylation Search

Non-enzymatic glycosylation search on identified proteins was performed using Proteome Discoverer 2.1 platform (ThermoFischer Scientific, Waltham, MA, USA) based on SEQUEST search engine and human protein database (70,726 entries, downloaded on March 2024 from UNIPROT website, https://www.uniprot.org/). Trypsin was set as a digestion enzyme; three missed cleavages were set at maximum; mass tolerances of 10 ppm for precursor ions and 0.05 Da for fragment ions were applied. A list of different dynamic modifications related to non-enzymatic glycosylations (Table 2) was set for Lys and Arg residues. A percolator node was used with target-decoy strategy to give final false discovery rates (FDR) at a Peptide Spectrum Matches (PSMs) level of 0.01 (strict) based on q-values, considering a maximum deltaCN of 0.05; a minimum peptide length of six amino acids and rank 1 were considered, and protein grouping and strict parsimony principle were applied. The identified protein list with PSMs values relative to glycated peptides was exported in .xlsx format for further evaluation of results.

### 4.12. Hierarchical Clustering

Starting from the list of identified proteins, average PSMs values for single subjects, resulting from technical replicates, was obtained. These lists were used to build a Hierarchical Clustering (HC) based on protein average PSMs values through JMP 15.2 software (SAS, Cary, NC, USA).

### 4.13. Label-Free Relative Quantification and DAP Extraction

Based on direct correlation between the spectral counts (SpC, also called PSMs) and the relative abundance of identified proteins, Linear Discriminant Analysis (LDA) was performed to identify features that discriminated Group I against Group II. 466 proteins were isolated and classified as Differentially Abundant Proteins (DAPs) among the two identified groups based on *p*-value < 0.05. In addition, DAve (Differential Average) and DCI (Differential Coefficient Index) indices of in-house developed MAProMa platform were used to process the average SpC (aSpC) corresponding to each analyzed subject group, identified by means of HC, as previously described [12]. The threshold values imposed were DAve > |0.2| and DCI > |5|. No internal standards were used in LC-MS/MS analyses for protein label-free quantification.

### 4.14. PPI Network

The 466 identified DAPs were used to extract a Protein-Protein Interaction (PPI) Network through Cytoscape 3.8 Software and its apps. Experimentally verified interactions were retrieved from several databases such as Prolink, DIP, KEGG and BIND, and only experimentally verified interactions with >0.15 score were retained. Specifically, the cutoff chosen for edge filtering guarantees that in scientific literature, there is at least an experiment that has verified the interaction between two given proteins that we assume interacts also in our system, if identified. STRING Enrichment App was used to emphasize functional modules in the PPI network, based on functionally organized gene ontology GO terms and pathway enrichment retrieved from KEGG. Concerning this point, it is important to underline that since the PPI network is analyzed from a functional point of view, a more stringent filtering criteria about these edges isn’t necessary, because functional enrichment doesn’t consider this information, and the retained PPIs assume a qualitative value. On the contrary, when the network topology is evaluated, thorough and more stringent filtering criteria are required [38,39].

### 4.15. FerrDB Enrichment

The list of 466 identified DAPs was submitted on FerrDB Database v2 (http://www.zhounan.org/ferrdb/current/) (accessed on 25 November 2024) [33], a database that dedicates to ferroptosis regulators and ferroptosis-disease associations, to evaluate possible enrichment of other ferroptosis-related terms in addition to the KEGG “ferroptosis” term found as enriched following the above-mentioned procedure. The list of enriched terms according to FerrDB was downloaded as a .jpg image.

### 4.16. Subjects’ Follow-Up

After a long follow-up period of about 6 years, patients were contacted by a phone interview to collect clinical information regarding the development of diabetes, hypertension, cardiovascular diseases (angina, myocardial infarct, heart failure), neoplasia, or SARS-CoV-2 clinical confirmed infection, in order to assess a correlation between proteomic profile and the long-term events in obese patients.

## 5. Conclusions

Our proteomic analysis of urine samples from 45 metabolically homogeneous obese subjects enabled the identification of individuals predisposed to a heightened pro-oxidant and inflammatory state through molecular stratification. This stratification represents a second level of redox-risk classification in this population, extending beyond the first-level, sex-based categorization. Importantly, our clustering approach allowed us to distinguish, within each sex, individuals exhibiting molecular profiles indicative of increased redox stress.

We acknowledge that these interpretations are preliminary and require validation through targeted studies designed to more comprehensively characterize the molecular profiles of homogeneous obese cohorts. Such studies should aim, on the one hand, to correlate proteomic signatures with routinely measured biochemical parameters and, on the other, to incorporate systematic longitudinal follow up, including repeated clinical and research assessments to evaluate their potential retrospective prognostic value in predicting the development of obesity-related pathologies.

## Figures and Tables

**Table 1:** Clinical, anthropometrical, and biochemical characteristics of the study population.

	ALL (N = 45)
Age, years	60 ± 5
Male gender, n (%)	25 (56%)
BMI, kg/m^2^	34 ± 3
BMI ≥ 35	18 (40%)
Waist circumference, cm	111 ± 10
Hypertension, n (%)	23 (51%)
Diabetes, n (%)	1 (2%)
Dyslipidemia, n (%)	16 (36%)
Glucose, mg/dL	101 ± 14
Insuline, (μU/mL)	15.7 ± 8.4
HOMA index	4.04 ± 2.49
Metabolic syndrome	18 (40%)
Creatinine, mg/dL	0.88 ± 0.22
AST, U/L	21 ± 6
ALT, U/L	24 ± 15
GGT, U/L	25 ± 25
Total cholesterol, mg/dL	206 ± 33
LDL cholesterol, mg/dL	133 ± 29
HDL cholesterol, mg/dL	50 ± 11
Triglycerides, mg/dL	116 ± 42
Fatty liver index	79 ± 14
Visceral adiposity index	4.00 ± 1.91

**Table 2:** Table of non-enzymatic glycosylation searched with Proteome Discoverer 2.1.

Modified Adduct	Abbreviation	Amminoacidic Residue
N_ε_-[5-(2,3,4-Trihydroxybutyl)-5-hydro-4imidazolon-2-yl]ornithine	3-DG-H1	R
Tetrahydropyrimidine	THP	R
Imidazolone B	IB	R
Argpyrimidine	ArgP	R
N_ε_-(5-Hydro-5-methyl-4-imidazolon-2-yl)ornithine	MG-H1	R
N_ε_-(5-Hydro-4-imidazolon-2-yl)ornithine	G-H1	R
Fructosyl-lysine	FL	K
Fructosyl-lysine-H_2_0	FL-1H_2_0	K
Fructosyl-lysine-2H_2_0	FL-2H_2_0	K
N_ε_-Carboxyethyl-lysine	CEL	K
N_ε_-Carboxymethyl-lysine	CML	K
Pyrraline	Pyr	K
1-Alkyl-2-formyl-3,4-glycosyl-pyrrole	AFGP	K and R

## Data Availability

Raw mass spectra acquired and processed for this paper are available at the link: ftp://massive-ftp.ucsd.edu/v12/MSV000101123/ (accessed on 13 March 2026). For peer-review, raw mass spectra are available at the following link: ftp://MSV000101123@massive-ftp.ucsd.edu (accessed on 13 March 2026), password: proteobesity.

## Supplementary Materials

The following supporting information can be downloaded at: https://www.mdpi.com/article/10.3390/ijms27114678/s1.
