# Supplementary material for: The Proteomics-Based Stratification of Obese Subjects Allows for a Second Selective Level Beyond Gender Classification
Source: Int J Mol Sci. 2026 May 22;27(11):4678. doi: 10.3390/ijms27114678 (PMC13257239; doi:10.3390/ijms27114678)
Supplement: Supplementary file 1 [file ijms-27-04678-s001.zip › Figures S1-S5.pdf]

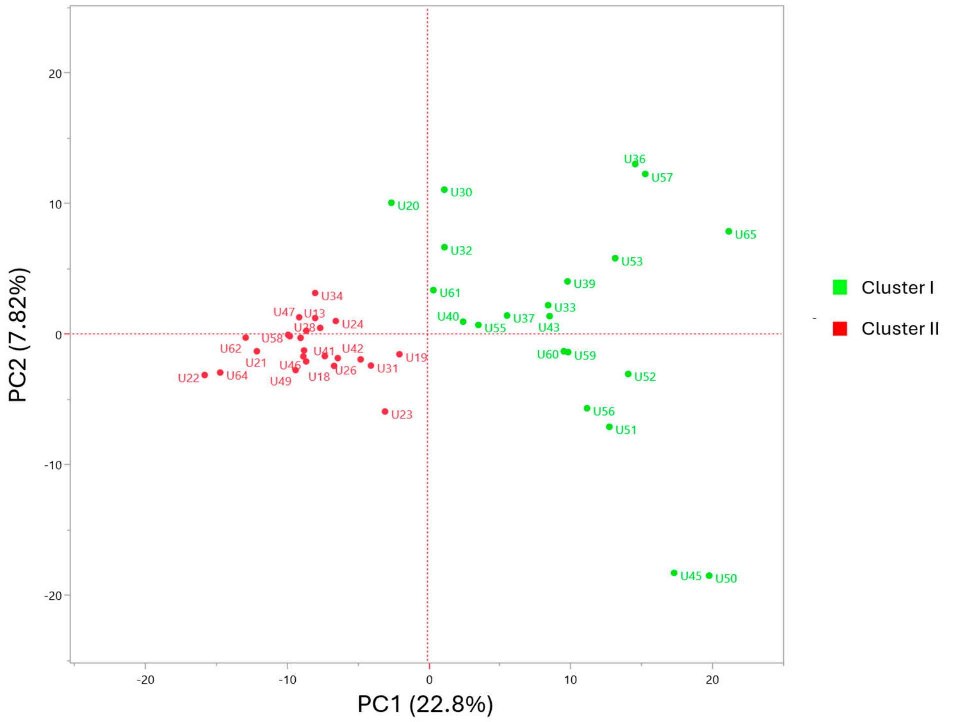

Figure S1. PCA (Principal Component Analysis) of the 45 subjects enrolled for this study. DAPs relative abundances were employed to perform PCA. Light green dots correspond to Cluster I subjects, while red dots correspond to Cluster II subjects.

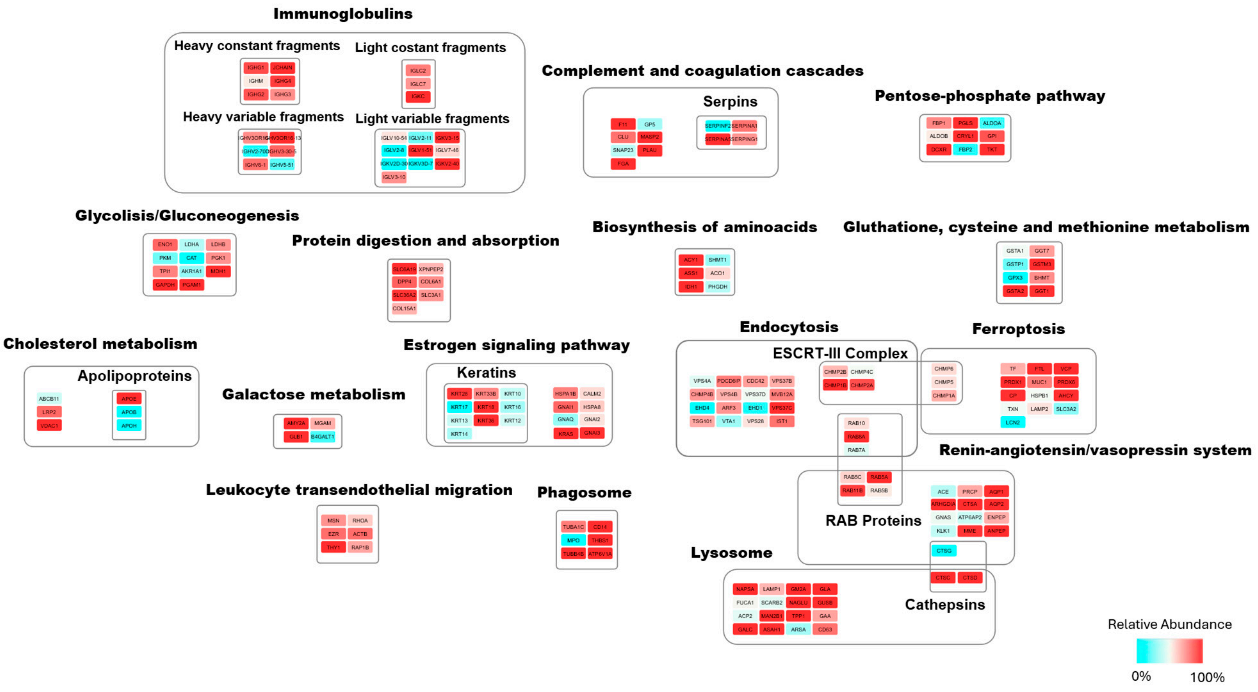

Figure S2. Protein-Protein Interaction (PPI) network of 466 DAPs identified for Group I Men subjects. Nodes in the network represent DAPs, while links among DAPs represent physical/functional interactions. DAPs are grouped according to KEGG functional annotation, and they are colored according to relative abundance in the four considered conditions (Men Gr. I, Women Gr. I, Men Gr. II and Women Gr. II), from light blue (no expression) to red (100% of relative abundance).

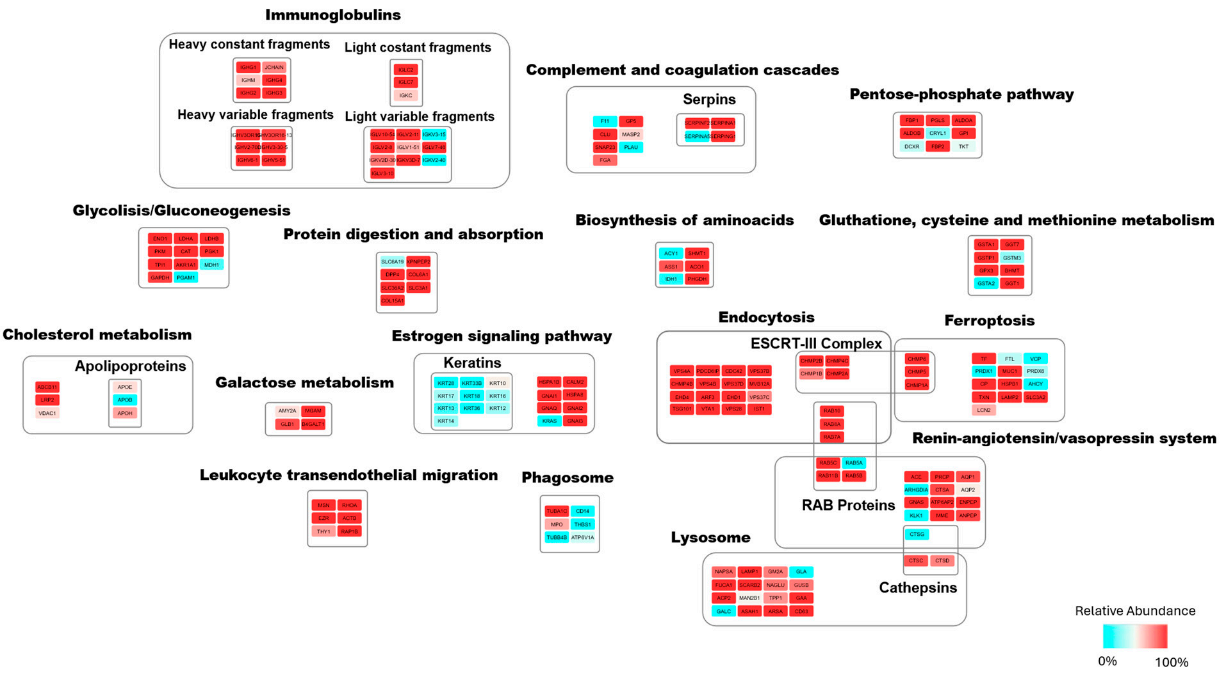

Figure S3. Protein-Protein Interaction (PPI) network of 466 DAPs identified for Group I Women subjects. Nodes in the network represent DAPs, while links among DAPs represent physical/functional interactions. DAPs are grouped according to KEGG functional annotation, and they are colored according to relative abundance in the four considered conditions (Men Gr. I, Women Gr. I, Men Gr. II and Women Gr. II), from light blue (no expression) to red (100% of relative abundance).

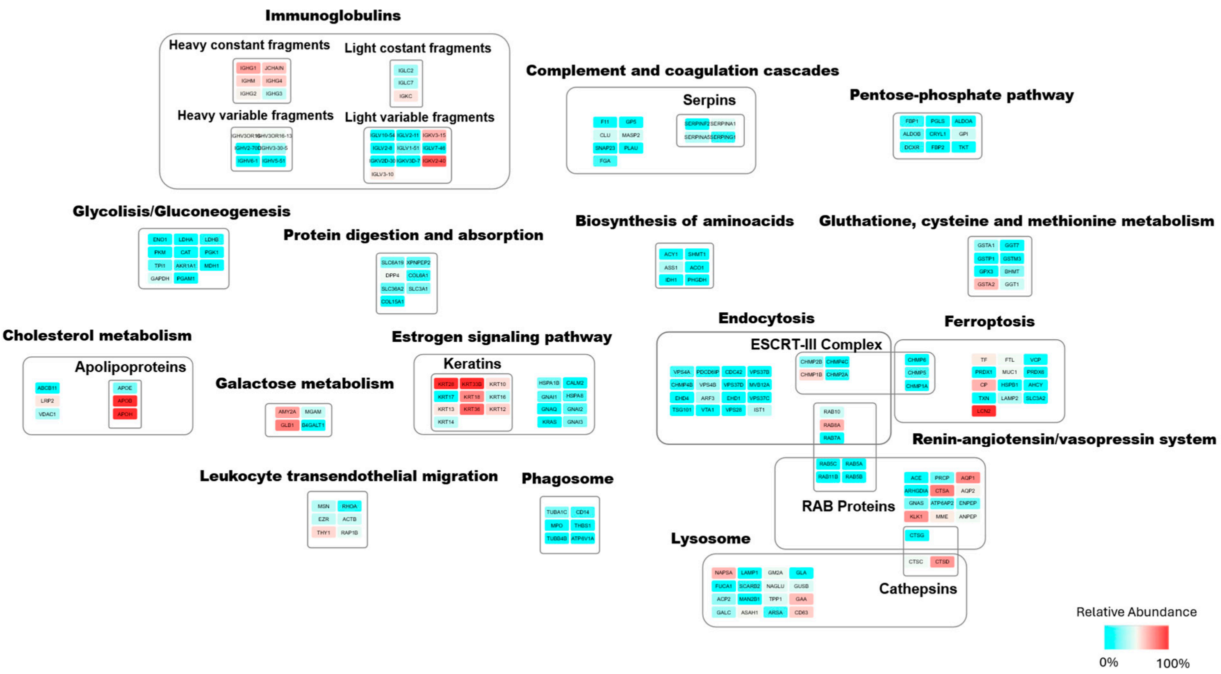

Figure S4. Protein-Protein Interaction (PPI) network of 466 DAPs identified for Group II Men subjects. Nodes in the network represent DAPs, while links among DAPs represent physical/functional interactions. DAPs are grouped according to KEGG functional annotation, and they are colored according to relative abundance in the four considered conditions (Men Gr. I, Women Gr. I, Men Gr. II and Women Gr. II), from light blue (no expression) to red (100% of relative abundance).

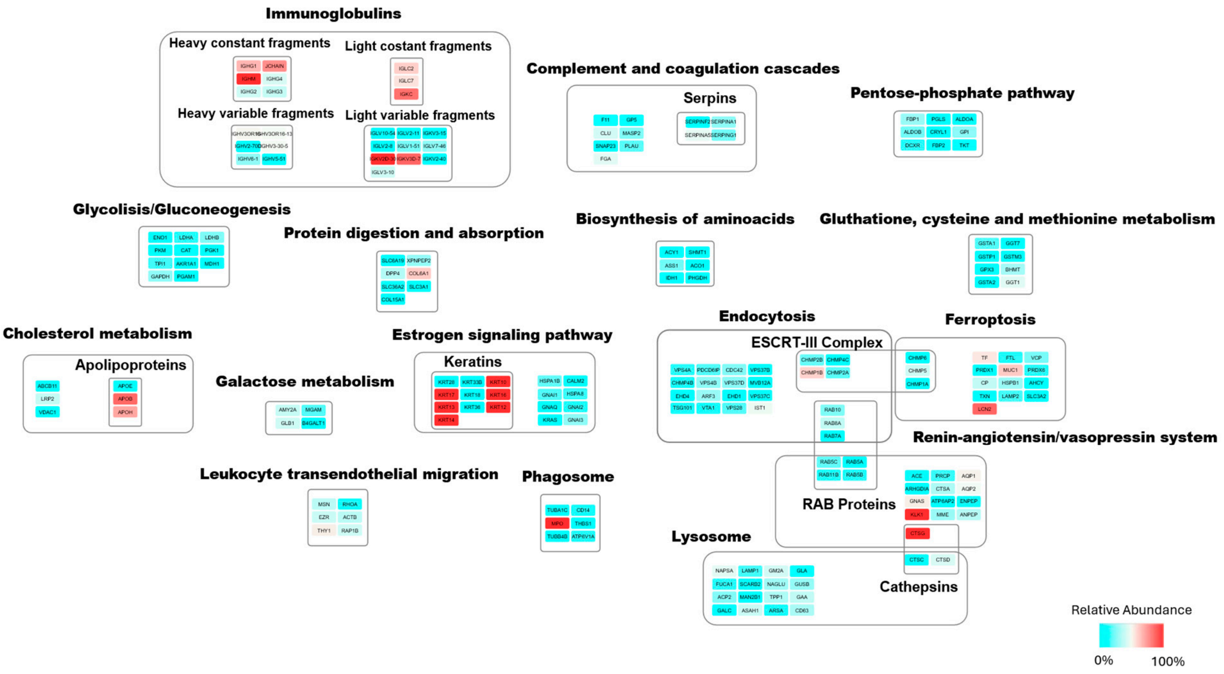

Figure S5. Protein-Protein Interaction (PPI) network of 466 DAPs identified for Group II Women subjects. Nodes in the network represent DAPs, while links among DAPs represent physical/functional interactions. DAPs are grouped according to KEGG functional annotation, and they are colored according to relative abundance in the four considered conditions (Men Gr. I, Women Gr. I, Men Gr. II and Women Gr. II), from light blue (no expression) to red (100% of relative abundance).
