# Supplementary material for: The Proteomics-Based Stratification of Obese Subjects Allows for a Second Selective Level Beyond Gender Classification
Source: Int J Mol Sci. 2026 May 22;27(11):4678. doi: 10.3390/ijms27114678 (PMC13257239; doi:10.3390/ijms27114678)
Supplement: Supplementary file 1 [file ijms-27-04678-s001.zip › Table S3.pdf]

Table S3. Clinical and biochemical characteristics of Cluster I and Cluster II.

	**Cluster I (N=21)**	**Cluster II (n=24)**	**P**
Age, years	59 ± 5	61 ± 5	0.068
BMI, kg/m^2	35 ± 3	34 ± 3	0.574
Lean Body Mass, (%)	58 ± 7	53 ± 6	**0.015**
Fat Body Mass, (%)	42 ± 7	47 ± 6	**0.016**
Male gender, n (%)	17 (81%)	8 (33%)	**0.002**
Hypertension, n (%)	12 (57%)	11 (46%)	0.554
Diabetes, n (%)	0 (0%)	1 (4%)	1.000
Dyslipidemia, n (%)	6 (29%)	10 (42%)	0.533
HbA1c, mmol/mol	37.1 ± 2.7	37.5 ± 4.8	0.936
HOMA Index	3.91 ± 1.81	4.16 ± 2.99	0.585
Metabolic syndrome	7(33%)	11 (46%)	0.393
Total cholesterol, mg/dl	210 ± 35	202 ± 33	0.385
LDL, mg/dl	140 ± 30	126 ± 27	0.119
HDL, mg/dl	50 ± 11	51 ± 11	0.640
Triglycerides, mg/dl	112 ± 45	120 ± 40	0.195
Fatty liver index	81 ± 13	77 ± 15	0.317
Visceral adiposity index	3.52 ± 1.85	4.42 ± 1.90	**0.037**
